# Reprogrammed Lipid Metabolism and the Lipid-Associated Hallmarks of Colorectal Cancer

**DOI:** 10.3390/cancers14153714

**Published:** 2022-07-29

**Authors:** Timothy Salita, Yepy H. Rustam, Dmitri Mouradov, Oliver M. Sieber, Gavin E. Reid

**Affiliations:** 1 Department of Biochemistry and Pharmacology, University of Melbourne, Parkville, VIC 3010, Australia; tsalita@student.unimelb.edu.au (T.S.); yrustam@student.unimelb.edu.au (Y.H.R.); 2 Personalized Oncology Division, The Walter and Eliza Hall Institute of Medical Research, Parkville, VIC 3052, Australia; mouradov.d@wehi.edu.au; 3 School of Chemistry, University of Melbourne, Melbourne, VIC 3010, Australia; 4 Bio21 Molecular Science & Biotechnology Institute, University of Melbourne, Parkville, VIC 3010, Australia

**Keywords:** colorectal cancer, CRC, lipids, lipid metabolism, metabolomics, cancer hallmarks

## Abstract

**Simple Summary:**

Colorectal cancer (CRC) is the third-most diagnosed cancer and the second-leading cause of cancer-related deaths worldwide. Limitations in early and accurate diagnosis of CRC gives rise to poor patient survival. Advancements in analytical techniques have improved our understanding of the cellular and metabolic changes occurring in CRC and potentiate avenues for improved diagnostic and therapeutic strategies. Lipids are metabolites with important biological functions; however, their role in CRC is poorly understood. Here, we provide an in-depth review of the recent literature concerning lipid alterations in CRC and propose eight lipid metabolism-associated hallmarks of CRC.

**Abstract:**

Lipids have diverse structures, with multifarious regulatory functions in membrane homeostasis and bioenergetic metabolism, in mediating functional protein–lipid and protein–protein interactions, as in cell signalling and proliferation. An increasing body of evidence supports the notion that aberrant lipid metabolism involving remodelling of cellular membrane structure and changes in energy homeostasis and signalling within cancer-associated pathways play a pivotal role in the onset, progression, and maintenance of colorectal cancer (CRC) and their tumorigenic properties. Recent advances in analytical lipidome analysis technologies have enabled the comprehensive identification and structural characterization of lipids and, consequently, our understanding of the role they play in tumour progression. However, despite progress in our understanding of cancer cell metabolism and lipidomics, the key lipid-associated changes in CRC have yet not been explicitly associated with the well-established ‘hallmarks of cancer’ defined by Hanahan and Weinberg. In this review, we summarize recent findings that highlight the role of reprogrammed lipid metabolism in CRC and use this growing body of evidence to propose eight lipid metabolism-associated hallmarks of colorectal cancer, and to emphasize their importance and linkages to the established cancer hallmarks.

## 1. Introduction

Colorectal cancer (CRC) is the third-most commonly diagnosed cancer worldwide and ranked second in cancer-related deaths in 2020 [1]. Despite this global burden, significant gaps remain in our knowledge of the detailed molecular changes that occur in CRC. Lipids comprise a large group of organic compounds that have a diverse range of cellular roles in activities including energy homeostasis, membrane biogenesis and structure, and cellular signalling [2]. Recent advances in lipidome analysis technologies have enabled a broader understanding of the physiological roles that lipids play in normal and diseased tissues and have contributed to the discovery of diagnostic and prognostic markers, and potential therapeutic targets [3,4,5]. To meet the demands of increased cellular proliferation, cancer cells coordinate the dysregulation of enzymes that control lipid metabolism [6]. However, lipid metabolism is also frequently dysregulated in a range of other diseases including obesity [7], diabetes [8], and neurodegeneration [9].

The ‘hallmarks of cancer’, first reported in 2000 by Hanahan and Weinberg [10], then revised and expanded in 2011 [11] and 2022 [12], describe the aberrant concomitant cellular processes and characteristic factors believed to unlock the uncontrollable proliferative nature of malignant cells [10,11]. These hallmarks now include avoiding growth suppressors, evading immune destruction, tumour-promoting inflammation, activating invasion and metastasis, stimulating angiogenesis, genome instability and mutation, deregulating cellular metabolism, sustaining proliferative signalling, unlocking phenotypic plasticity, non-mutational epigenetic reprogramming, senescent cells, and polymorphic microbiomes [12]. The improved understanding of cancer cell metabolism in recent years supports the notion that reprogrammed lipid metabolism is a common characteristic of cancers [7,13]. Here, we review recent findings that highlight the interplay between altered lipid metabolism in CRC tumour development and maintenance, which supports the notion that altered lipid metabolism is an underlying characteristic of the hallmarks of this disease.

### 1.1. Clinical Presentation of CRC

CRC encompasses malignancies originating from colonic and rectal mucosal tissues and suffers from a high mortality rate attributable to late-stage disease diagnosis and metastatic progression [14]. Approximately 75% of all CRC cases occur sporadically as a result of acquired genetic and epigenetic somatic modifications [15].

Early detection of CRC improves patient prognosis, treatment success, and reduces morbidity and mortality [14]. CRC screening is commonly achieved with faecal occult blood tests that examine stool samples for the presence of haem, an early indicator of colon and rectal abnormalities and lesions [16]. Colonoscopy is the most common method of diagnosing CRC, with computed tomography (CT), magnetic resonance imaging (MRI), and positron emission computed tomography (PET) commonly used to assess the spread of CRC neoplasms. Surgical removal of affected areas of the bowel by colectomy is the primary treatment for colorectal tumours. In addition to surgery, other treatments may include chemotherapy, radiotherapy, targeted drug therapy, and immunotherapy.

Tumour pathological staging and grading post-surgical resection may be complemented by tumour molecular classification [17]. Major molecular classification approaches for CRC include (i) chromosome instability (CIN), (ii) hypermutation due to DNA mismatch-repair deficiency (dMMR) or POLE mutation, (iii) CpG island methylator phenotype (CIMP), and (iv) transcriptomic consensus molecular subtype (CMS) [18,19]. CIN tumours exhibit chromosomal abnormalities and aneuploidy and comprise ~70% of all CRC [18]. CIN-CRCs are strongly associated with the classic adenoma–carcinoma sequence of tumorigenesis with mutations in *APC*, *KRAS*, *PI3KCA*, and *TP53* genes. Hypermutated CRCs with dMMR account for approximately 15% of all CRC and are characterized by slippage in short DNA tandem repeats, termed microsatellite instability (MSI) [20]. MSI in sporadic CRCs is commonly caused by epigenetic silencing of the MMR gene MLH1, and it tends to co-occur with CIMP and *BRAF* mutations. On the other hand, hypermutated tumours with POLE mutation exhibit a significant increase in nucleotide substitutions and account for 1–2% of CRC [21]. Lastly, CMS classification categorizes tumours into four groups based on their gene expression landscape [19]. The diversity in oncogenic pathways, mutations, and abnormal events that occur in CRC makes it a highly heterogeneous disease with implications for prognosis and therapeutic targeting.

### 1.2. Lipidomics for Characterizing Lipid Structures

**Figure 1:**
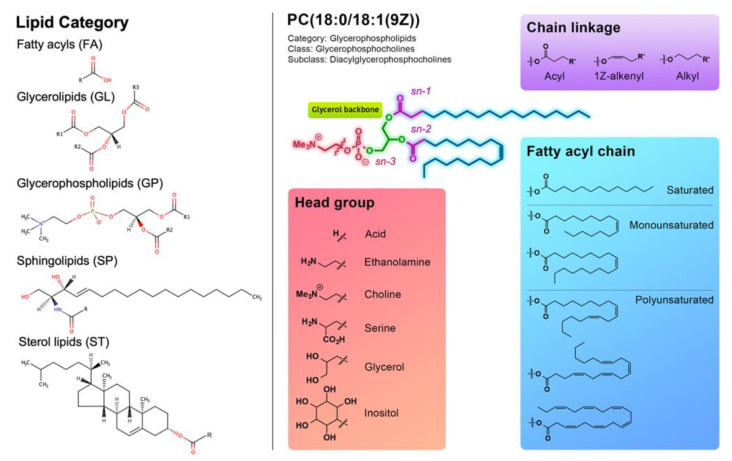
Common structural features of lipid molecules. Lipid categorization, classification, and structural features according to LIPID MAPS consortium [22]. Five lipid categories clinically relevant to CRC are shown: FA, fatty acyls; GL, glycerolipids; GP, glycerophospholipids; SP, sphingolipids; ST, sterols. Structural features include functional head groups, fatty acyl chain types, and chain linkage diversity.

Lipids are broadly defined as hydrophobic or amphipathic small molecules that may originate entirely or in part by carbanion-based condensations of thioesters (fatty acyls, glycerolipids, glycerophospholipids, sphingolipids, saccharolipids, and polyketides) and/or by carbocation-based condensations of isoprene units (prenol lipids and sterol lipids) [22]. The structural and functional diversity of lipids is mediated by a range of structural backbone components and functional headgroups, as well as the chain length of fatty acyl (FA) groups and the number and position(s) of carbon–carbon (C=C) double bond unsaturation(s), and acyl, alkyl and alkenyl chain-linkage type (Figure 1) [2].

Lipid molecules fall into eight distinct categories, five of which have been implicated in the pathogenesis of CRC; FA, glycerolipids (GL), glycerophospholipids (GP), sphingolipids (SP), and sterol lipids (ST) [22]. The remaining categories comprise prenol lipids (PR), saccharolipids (SL), and polyketides (PK). Human cellular membranes are largely composed of GPs, SPs and STs [23]. Lipids are further classified into classes and sub-classes based on their distinct structural motifs and physiochemical properties [22]. The complexity and molecular diversity of the structures of lipids within each category, class, and sub-class is highlighted by the LIPID MAPS Structure Database (LMSD), which compiles experimentally identified (i.e., curated) and computationally generated lipids complete with annotations, structural information, analytical tools, and research data. Overall, as of April 2022, there are 25,426 individually curated and 22,028 computationally generated lipid species (47,454 total) recorded in the LMSD.

The diverse functions of lipids are highly dependent on their structures, physiological concentrations, and intra- and inter-cellular spatial and temporal distributions. FAs are important constituents of biological membrane-associated lipids and are derived from de novo FA synthesis and remodelling or via exogenous FA uptake mechanisms [23,24]. In addition to maintaining cell wall integrity, GPs are also associated with signal transduction and cell motility processes [25,26]. GLs such as triacylglycerides (TGs) serve as primary energy stores of FAs in most living organisms [27], while the TG precursors, diacylglycerides (DG) have roles in signalling mechanisms such as protein export and membrane fusion [28]. SPs such as sphingosine and ceramide have fundamental physiological functions in cell survival and angiogenesis mechanisms [6], whereas the function of sterol-derived cholesterol lipids include the regulation of signal transduction pathways via membranous lipid raft microdomains [2].

Lipidomics, as an integral but experimentally distinct discipline within the field of metabolomics, aims to identify, characterize and quantify the structure and function of lipids and their associated regulatory mechanisms involved in health and disease. Lipidomics can also provide insights into disease-associated or disease-specific lipid homeostasis dysregulation and therefore has significant potential for use in biomarker and therapeutic discovery strategies [5,29]. Cancer biomarker discovery has utilized lipidomics in recent years due to rapid advances in methodologies and technologies for lipid identification, characterization and quantitative analysis, in particular using mass spectrometry (MS) and MS-imaging techniques [30]. Although a wide range of analysis strategies exist, MS based lipidomic analysis workflows are typically composed of a series of steps including sample preparation followed by chromatographic separation or direct ‘shotgun’ infusion typically with electrospray ionization (ESI), or matrix-assisted laser desorption ionization (MALDI) (particularly for imaging applications), followed by data acquisition using either ‘targeted’ or ‘untargeted’ MS and tandem mass spectrometry (MS/MS) methods in low-, high-, or ultrahigh-resolution mass analyzers. Finally, software-based database searching is used for lipid identification, followed by statistical and bioinformatic analysis [29]. Stable isotope labelled lipids for each lipid class or sub-class are typically included as internal standards to enable quantitation of individual endogenous lipid concentrations. The resultant lipidomic data sets are increasingly integrated with those generated from complementary genomic, transcriptomic, proteomic and metabolomic studies to derive more detailed insights into the underlying biological phenotype. Detailed accounts of recent advances in lipidomics technologies have been reviewed by Rustam et al. [29] and Wolrab et al. [5], while recent advances in lipidomics for clinical applications have been reviewed by Meikle et al. [31].

## 2. The Role of Lipids in CRC

### 2.1. Fatty Acyls

FAs serve as important structural components of cell membrane-associated lipids, as substrates for energy production, and as secondary messengers in signalling pathways (Table 1) [32]. FAs can be classified based on their carbon chain length, and in the degree and position of their C=C double bond unsaturations, e.g., saturated FA (SFA), mono-unsaturated FA (MUFA), poly-unsaturated FA (PUFA), short-chain FA (SCFA), medium-chain FA (MCFA), long-chain FA (LCFA), and very long-chain FA (VLCFA) [33]. De novo lipogenesis commences with FA synthesis to produce FA(16:0) (palmitic acid), while exogenous or dietary lipids are metabolized to produce essential FAs: FA(18:3(n-3)), FA(18:2(n-6)), and other long-chain PUFAs [34]. Chain elongation and C=C desaturation of FAs via a range of elongase (i.e., elongation of very long-chain fatty acids (ELOVLs)) and desaturase (i.e., stearoyl-coA desaturase (SCD) and fatty acid desaturase (FADS)) enzymes, produce a diverse spectrum of FAs that serve as resources for GL, GP, SP and ST lipid synthesis [35]. Alternations in FA structures play a key role in regulating membrane lipid physico-chemical properties and dynamics [36,37]. For example, lipids containing linear SCFAs form membranes with lower viscosity than their dynamic MUFA, while PUFA-containing counterparts increase membrane fluidity [38].

During cancer transformation, a switch in energy metabolism, termed the Warburg effect, describes the ability of cancer cells to preferentially undergo aerobic glycolysis over oxidative phosphorylation to produce energy [85]. This metabolic change towards preferential glucose and glutamine uptake enhances lipid metabolism via increasing exogenous dietary lipids and lipoproteins, or upregulating cholesterol and de novo lipid biosynthesis [2]. By reprogramming lipid metabolism and cellular energetics, a known cancer hallmark [11], cancer cells accumulate beneficial metabolic products that favour their growth and survival. For example, fatty acid synthase (FASN) generates fatty acids from malonyl-CoA and acetyl-CoA via de novo FA synthesis and is upregulated in colorectal cancer [24,76]. A recent study of CRC tissues showed that FASN was upregulated in tumours compared to normal mucosa [58]. Furthermore, the inhibition of FASN in CRC-associated fibroblasts and the inhibition of FA uptake in CRC cells has been shown to attenuate cell migration [86].

Alterations in the content of various FAs has been associated with an increased risk of diseases including CRC. For instance, an increase in the level of plasma SFAs has been associated with an increased risk of CRC [87,88], and a shift towards SFAs rather than PUFAs in polar plasma lipids was reported in CRC patients [44]. Enhanced activity of ELOVL enzymes in CRC tissue has been shown to lead to an accumulation of VLCFAs, such as FA(26:0) (hexacosanoic acid) [61]. Additionally, ELOVL5 mRNA expression has been shown to be upregulated in CRC tumours compared to normal matched mucosa [58]. The elevation of saturated and monounsaturated fatty acyl chain content in membrane lipids has also been associated with upregulated de novo lipogenesis in CRC cell lines [89]. Since C=C double bonds are susceptible to oxidative attack, a high content of cell membrane SFAs may reduce cell susceptibility to free radical damage and drug penetration [89]. In plasma, stearic acid FA(18:0) SFA and ω-6 arachidonic acid FA(20:4) PUFA have both been positively associated with CRC risk in a genotype Mendelian randomisation analysis [90]. Lastly, a recent study showed colorectal tumours contained elevated levels of FA(26:0) and FA(28:0) compared to normal mucosa [91].

In addition to FA elongation, FAs can also be metabolized by SCD and FADS enzymes to produce unsaturated Fas [35]. SCD1 is responsible for synthesizing MUFAs from Δ9 SFAs, from which PUFAs can be generated by subsequent desaturation of MUFAs by FADS1 or FADS2 [52]. In CRC tissues, one study showed both FADS1 and FADS2 mRNA expression was upregulated in tumours [58], and increased SCD1 expression was reported in another study [53]. This increase in SCD1 was associated with an accumulation of MUFAs, increased epithelial–mesenchymal transition (EMT), and inhibition of the tumour suppressor PTEN, which promoted CRC metastasis. Furthermore, various cancer cells resistant to pharmacological SCD inhibition have been shown to utilize an alternative FA desaturase pathway that produced *cis-6* SFA(16:0) (sapienic acid) [92]. By doing so, SCD-independent cancer cells evade the growth inhibitory effect of SCD inhibition. Other MUFA species have also been reported to be associated with cancer. For example, increased vaccenic acid (ω-7 FA(18:1)) is believed to be associated with the upregulation of ELOVL5 and ELOVL6 and may indicate increased de novo lipogenesis as a result of oncogenic KRAS and Sterol Regulatory Element-Binding Proteins (SREBP1 and SREBP2) activation [52,54].

PUFAs can be classified into two categories, ω-6 PUFAs and ω-3 PUFAs, that exhibit pro-inflammatory and anti-inflammatory (or pro-resolving) effects, respectively [93]. Despite conflicting roles in inflammation, a recent lipidomic study of CRC tissues revealed that both ω-6 PUFA FA(20:4) (arachidonic acid (AA)) and ω-3 PUFAs such as FA(20:5) (eicosapentanoic acid (EPA)) and FA(22:6) (docosahexanoic acid (DHA)) were up-regulated in CRC compared to normal tissue [55]. The ω-6 FA(22:4) (docosatetraenoic acid) was also reported to be accumulated in plasma from early stage CRC patients compared to control [56]. In addition to accumulated PUFAs in CRC tissues compared with normal mucosa, it was also reported that CRC cells upregulate the uptake of PUFA from culture medium [57]. Fatty acid-derived prostaglandins have a direct bioactive role in mediating inflammatory pathways [79]. For example, Prostaglandin E2 (PGE2), synthesized from the PUFA arachidonic acid by cyclooxygenase-2 (COX2), induces inflammation and promotes tumour progression [79]. Thus, with distinguishable functions, various PUFAs have been proposed as biomarkers for CRC [94].

### 2.2. Glycerolipids

GLs comprise a group of neutral lipids that have predominant roles in energy storage and membrane lipid biosynthesis (Table 1) [27]. Monoacylglyceride (MG), DG and TG lipids predominantly constitute this lipid category and are defined by containing one, two, or three FAs, respectively, attached to a glycerol backbone via ester or ether bonds (Figure 1). GL synthesis involves the sequential acylation of MG to DG by monoacylglycerol o-acyltransferase (MOGAT) enzymes, and DG to TG by diacylglycerol o-acyltransferase (DGAT) enzymes [95]. Alternatively, TGs can also be synthesized via the glycerol-3-phosphate pathway, known as the Kennedy pathway [27]. Glycerolipid degradation involves hydrolysis of TG to DG by adipose triglyceride lipase (ATGL), followed by DG to MG by hormone sensitive lipase (HSL), and lastly the breakdown of MG to FA and glycerol moieties by monoacylglycerol lipase (MGL). In CRC cells, ATGL over-expression has been shown to promote cell proliferation, whilst its inhibition induced CRC cell apoptosis [80]. Additionally, the expression of MGL mRNA and protein were significantly reduced in CRC tissues compared to patient-matched normal tissue, MGL over-expression attenuated cell colony formation, and MGL knock-down increased Akt activation, which suggests a role for MGL in CRC tumorigenesis [81].

Variations in the structure of TGs (i.e., the number of acyl chains, acyl chain length and the degree of unsaturation) influence their physical and chemical properties [96]. The metabolism of TGs to release FA for energy or lipid synthesis results in activation of FAs into metabolic acyl-CoA [60]. For instance, LCFAs are synthesized and activated by long-chain acyl-CoA synthetases (ACSLs), which are also commonly dysregulated in cancer [60]. The over-expression of the ACSL4 isoform has been identified as a marker for poor patient survival in stage II CRC [82]. Furthermore, an accumulation of both VLCFAs in TGs, as well as non-esterified VLCFAs has been observed in CRC tissues. This accumulation was associated with increased ELOVL1 expression whereby its inhibition decreased both VLCFA-TG and non-esterified VLCFA levels [62]. Changes in FA metabolism enzymes have also been reported in prostate cancer, where elevated expression of ACSL1 was associated with serum acyl-CoA levels, which promoted cancer progression via augmented β-oxidation and lipogenesis [97]. ACSL1 knock-down has been shown to result in decreased prostate cancer cell migration and proliferation in vitro and inhibited the growth of prostate cancer xenograft tumours in vivo [97].

TGs are stored in lipid droplets (LD), which are dynamic organelles characterized by a phospholipid monolayer containing lipid metabolism-associated proteins, and a neutral lipid core consisting of TGs and sterol-esters [98]. Cancer cells can accumulate TGs as a consequence of upregulated FA uptake and lipogenic enzymes, which ultimately serves as a reservoir of FAs that can be metabolized for bioenergetic β-oxidation or membrane biosynthesis [83]. As a result of TG upregulation, lipid droplet accumulation has, in recent years, become an emerging feature of cancer cells [59]. CRC stem cells exhibit accumulation of lipid droplets compared to normal colon epithelial cells [99]. The increase in lipid droplet number and size in CRC cells was demonstrated to be directly correlated with increased expression of lysophosphatidylcholine acyltransferase-2 (LPCAT2) [59]. In the CRC cell line CACO-2, lipid droplets have been shown to co-localize with COX2, and prostaglandin E synthase (PGES) proteins [100]. Increased abundance of lipid droplets was further observed with a concomitant increase in levels of PGE2 and COX2 expression. Using the FAS inhibitor, C75, on CACO-2 cells resulted in decreased lipid droplet formation, PGE2 synthesis, and cell proliferation [100], which suggests that lipid droplets function as regulatory reservoirs for prostaglandin formation and, thus, mediate inflammatory pathways essential in cancer cell metabolism.

In addition to dysregulation of glycerolipid metabolism enzymes and lipid droplet levels, cancer cells also exhibit alterations in specific TG lipid species. For instance, lipidomic analysis of patient-derived CRC tissues (*n* = 41) demonstrated a decrease in TGs with <53 carbon index (i.e., TG(48:2), TG(50:2), TG(50:3), and TG(52:3)) and an increase in TGs with >56 carbon index (i.e., TG(56:4), TG(56:5), and TG(56:6)) relative to non-diseased tissue [58]. This study further reported TG signatures as a potential discriminant in differentiating tumour from non-cancerous tissue. Conversely to the accumulation of LDs, other recent studies have reported decreased TG levels in patient-derived CRC tissues compared to normal mucosa [39,55]. Mika et al. reported reduced levels of TG in a study of 25 CRC tissue and patient-matched controls using ^1^H-nuclear magnetic resonance (^1^H-NMR) methods [55], while Pakiet et al. observed decreases in 21 TG, 8 DG, and one MG lipid species and decreased overall TG content in an LC-MS study of 10 patient-derived CRC tissues compared to normal mucosa [39]. Despite numerous reports of TG alterations in CRC tissues, one study found no significant differences in TGs between CRC tumours and normal mucosa [45]. TG metabolism supplies cells with FAs that promote cell growth via membrane and energy biogenesis [101]. Despite reports that LDs are upregulated in CRC cells [39,99,100] and important for cell proliferation [102], increasing evidence suggests that an accumulation of LDs in CRC cells occurs paradoxically with a decrease in TG content in CRC tissues [39,55].

Whilst numerous studies have explored changes in TG metabolism between CRC and non-cancerous tissues, lipidome analysis of isogenic primary SW480 and metastatic SW620 CRC cells provided insight into TG metabolism associated with CRC metastasis [43]. For instance, this study showed an increased proportion of TG content in SW620 cells compared to SW480 cells, which was linked to the function of TG lipids as a source of FAs necessary to maintain proliferation [43]. Other changes associated with metastatic SW620 CRC cells include an accumulation of ether-containing TG species such as TG-O, a concomitant decrease in corresponding non-ether triacyl-TGs, an increased abundance of long chain (>C56) TGs, and a decrease in odd-chain TG lipids [43]. As such, Fhaner et al. linked these alterations to upregulated de novo lipogenesis and metastatic capacity of malignant cells [43].

### 2.3. Sphingolipids

SPs are a category of lipids that contain a sphingoid base, an amide-linked fatty acid, and functional headgroups that can vary in structure from a hydrogen moiety, to phosphate, glucose, FAs, phosphocholine, galactose, or complex glycans [103]. Sphingolipids are amongst the important components of cell membranes that regulate cancer cellular signalling pathways involved in growth, metastasis, migration, and proliferation (Table 1) [70]. Cancer cells frequently show reprograming of sphingolipid metabolism including the downregulation of ceramides and sphingosine-1-phosphate (S1P) [70], and alterations in the metabolism of complex glycosphingolipids (GSL) [104].

Ceramides typically comprise a sphingosine C18 long chain base (LCB) and amide-linked FA chain of varying lengths [70,105]. Ceramide synthases 1 to 6 (CERS1-6) catalyze the de novo synthesis of dihydroceramide from sphinganine, which is then desaturated by dihydroceramide desaturase 1 (DEGS1) to produce ceramide [70]. Various CERS enzymes catalyze the production of ceramides with varying fatty acyl lengths. CERS1/4 produces C18-C20, CERS2 generates C22-C24, CERS3 synthesizes C28-32, and CERS5/6 produces C14-C16 ceramides [70]. CERS1-6 have also been demonstrated to produce LCBs containing C18 and C20 Fas [70]. Ceramides are also generated by sphingomyelinases (SMases) that hydrolyze sphingomyelin (SM) or by glucosylceramidase (GlcCDase) and galactosylceramidase (GCDase) that metabolize glucosylceramide and galactosylceramide, respectively [70].

Ceramides with varying FA chains have different biological roles that may be attributed to spatial localisation and intracellular trafficking [70], and are also considered to have anti-proliferative effects via regulation of cell cycle arrest and apoptosis [106,107]. However, various ceramides harbour conflicting functions, for instance LCFA- or VLCFA-containing ceramides exhibit pro-apoptotic and anti-apoptotic properties, respectively. A lipidomic study of isogenic primary and metastatic CRC cell lines showed increased proportions of ceramides and SM in metastatic SW620 cells relative to its primary SW480 counterpart [43]. SW620 cells contained increased levels of Cer(40:1), Cer(42:2), SM(40:1), SM(40:2), SM(41:1), SM(41:2), SM(42:1), SM(42:2), and SM(42:3) lipids relative to SW480 [43]. Despite overall increased proportions of both Cer and SM, metastatic SW620 cells showed decreased C16-containing SM(42:1) and Cer(42:1) levels compared to SW480, which was linked to the role of C16-containing sphingolipid function in apoptotic resistance [43]. Additional decreased lipids in SW620 cells include Cer(42:1), Cer(34:1), SM(32:1), SM(34:2), SM(36:1), and SM(36:2) [43]. Furthermore, an increase in SM(34:1), and decrease in SM(36:1) and SM(40:1) has also been reported from lipidomic analysis of CRC patient-derived tissues relative to normal-matched tissue [58].

Ceramides may also function in stress-induced signalling pathways and have been implicated in Stearoyl-CoA Desaturase 1 (SCD-1)-mediated apoptosis [50,51]. The elevation of saturated ceramides (i.e., C16- to C24-containing Cer) along with an increase in reactive oxygen species (ROS) in the CRC cell line LOVO treated with SCD-1 inhibitor has been shown to result in the attenuation in cell proliferation and increased apoptosis due to mitochondrial dysfunction. Tumour growth inhibition of mouse xenografts treated with SCD-1 inhibitor could be reversed by administration of a ceramide biosynthesis inhibitor [42]. Different ceramides have also been implicated as potential CRC biomarkers. For example, increased C16-, C24-, and C24:1-containing Cer and decreased C18- and C20-containing Cer in tumour tissue relative to normal tissue [42,108]. Another study reported decreased levels of Cer(36:1) and Cer(38:1) in CRC tumours compared to normal tissue, with a propensity for long-chain saturated ceramide species in CRC tumours of the right colon [58]. Additionally, CERS5 was found to be upregulated in CRC tissue and was associated with poor patient survival [71]. Cancer models have previously demonstrated alterations in ceramide synthetic and degradative enzymes that favour the loss of ceramides [75]. For instance, alkaline-sphingomyelinase (Alk-SMase), which generates ceramide from SM, is down-regulated in tissues of patients with sporadic colon adenoma [73].

Changes to ceramide metabolism has also been implicated in other cancers. For example, in a papillary thyroid cancer cell line model, the over-expression of CERS2 has been shown to induce apoptosis and G0/G1 cell cycle arrest [109]. In contrast, gene silencing of CERS6 resulted in decreased cell migration and invasion capabilities in vitro and metastatic potential in vivo of non-small cell lung carcinoma models [110]. Additionally, the knockdown of CERS1 in an oral cancer model resulted in reduced in vitro cell migration, proliferation, and invasion [111]. This study demonstrated that CERS1 knockdown was associated with endoplasmic reticulum (ER) stress and upregulated vascular endothelial growth factor A (VEGFA), which promoted oral cancer aggressiveness and chemotherapy drug resistance. These studies demonstrate the multifarious physiological role of ceramides.

Exposure to ursolic acid resulted in increased Alk-SMase activity in HT29 CRC cells and decreased tumour growth and activated caspase resulting in apoptosis [74]. Furthermore, ceramide synthase-6 (CerS6), which produces C16 ceramides responsible for stimulating tumour necrosis factor-related apoptosis-inducing ligand (TRAIL) response, is commonly down-regulated in CRC [70]. Enhanced pro-apoptotic caspase activation and cytochrome *c* release induced cell death in colon cancer cells via increased ceramides using ceramide analogues or by inhibiting ceramidases (CDases), enzymes responsible for generating sphingosine from ceramide [72]. Lastly, P53-dependent upregulation of CERS5 has been shown to enhance the chemosensitivity of colon cancer cells to 5-fluorouracil (5-FU) [112]. Among other sphingolipids altered in CRC, a recent study demonstrated changes in the metabolism of complex GSL glycans [104]. For instance, poorly differentiated cells contained a differential glycan profile to that of differentiated colon-like cells [104]. It was reported that colon-like cells highly expressed (neo)lacto-series, (sialyl)-Lewis^A/X^, and Lewis^B/Y^ antigen-containing GSLs compared to undifferentiated cells [104].

S1P is synthesized by the transfer of a phosphate headgroup onto sphingosine catalyzed by sphingosine kinase 1 or 2 isoforms (SK1 and SK2) [105]. S1Ps comprise a sphingoid base backbone bearing a C=C bond at the *n-14* position. S1Ps can subsequently be degraded by S1P lyase to form intermediates used in glycerolipid metabolism, or dephosphorylated by S1P-phosphatase 1 or 2 (SPP1 or SPP2) in the endoplasmic reticulum [41]. S1Ps binds and activates S1P-specific G-protein coupled receptors, S1PR1 and S1PR2, to regulate lymphocyte trafficking, cytoskeleton arrangements, and vascular homeostasis [41]. In CRC, agonist-mediated activation of SK1 and the accumulation of S1P second messenger have been shown to augment cell growth, survival and neoangiogenesis [106,107]. SK1 and SK2 enzymes are often over-expressed in CRC, with higher levels of SK1 correlating with metastatic CRC progression [75]. S1P can also accumulate via down-regulation of the S1P metabolic enzymes, S1P-lyase (SPL) and S1P-phosphatase (SPP) [75]. The accumulation of S1P leads to several effects beneficial to CRC cells, such as inhibition of ceramide-induced apoptosis for cell survival, promotion of angiogenesis via S1P-receptor activation, and promotion tumour-supporting inflammation via activation of NF-_Κ_B [113]. S1P can also stimulate cyclo-oxygenase 2 (COX2) activation leading to the generation of pro-survival prostaglandin E2 (PGE2), which mediates apoptotic resistance, proliferation, invasion and metastasis, and angiogenesis [79].

### 2.4. Sterols

Cholesterol is a major component of cell membranes, accounting for 30–50% membrane lipids, and its synthesis is frequently upregulated in cancer (Table 1) [2]. Alongside sphingolipids, cholesterols are also important components of membranous lipid rafts, which serve as platforms in cell signalling transduction pathways involved in cancer proliferation, invasiveness, metastasis, and chemoresistance [114,115]. Cholesterols are amphiphilic lipids comprising a hydrophobic steroid body consisting of three six-carbon rings and one five-carbon ring, and a hydrophilic hydroxyl headgroup (Figure 1) [116]. De novo cholesterol synthesis, also known as the mevalonate pathway, involves the precursor acetyl-CoA and rate-limiting enzymes 3-hydroxy-3-methylglutaryl coenzyme A reductase (HMGCR) and squalene monooxygenase (SM), among other enzymes. After a series of approximately 30 reactions, cholesterol is produced from lanosterol, and can be further esterified into lipid droplets as cholesteryl esters (CE) [117]. Cholesterol metabolism also encompasses the conversion of cholesterol to oxysterols, bile acid, and steroid hormones [117]. In addition to incorporation of CE lipids into LDs, cholesterols are primarily located in lipid membrane domains [117].

The synthesis and metabolism of cholesterol is regulated by Sterol Regulatory Element Binding Protein 1 and 2 (SREBP1 and SREBP2), which are tightly regulated by Insig-1 and SREBP-cleavage activating protein (SCAP) complexes that function in response to cholesterol levels [118]. Decreased levels of cholesterol results in the dissociation of Insig-1 from SCAP, and the S1P-mediated activation and nuclear translocation of SREBPs for FA and cholesterol synthesis [118]. The strong affinity of cholesterol with less saturated and saturated hydrocarbons facilitates the formation and stability of lipid rafts, and further reduces membrane permeability [119]. By doing so, the stable lipid rafts orchestrate the effectiveness of cell signalling networks. Cholesterol has diverse cellular functions including regulation of apoptosis and signalling such as activation of mitogen-activated protein kinase (MAPK), mammalian target of rapamycin (mTOR), β-catenin, c-Met and c-Src [118,120].

Membranous lipid rafts comprising sphingolipids and cholesterol are commonly upregulated in cancer, activating downstream proliferative signalling effector proteins such as AKT or Ras [121]. In cancer, mutant P53 can further promote upregulation of lipogenic enzymes involved in FA, cholesterol, TG, and GP synthesis by modulating expression of SREBPs [122]. Our understanding of the roles of cholesterol in lipid rafts is broadening, and since lipid rafts play important roles in mediating cell signalling cascades, cholesterol has emerged as a promising target in cancer therapeutics. In a recent study, cholesterol demonstrated inhibitory effects in CRC cancer cell apoptosis and promoted cellular proliferation [64]. PIM3, a serine/threonine kinase, was demonstrated to phosphorylate and inhibit proteins important in promoting CRC cell apoptosis, and inhibition of PIM3 with miR-33a mitigated the effect of cholesterol on CRC cells. Furthermore, upregulated expression of ATP-binding cassette transporter (ABCA1) was associated with poor CRC prognosis via promoting tumour progression through processes that involve cholesterol transport [66]. Oncogenic expression or mutations in *KRAS* and *PIK3CA* activates SREBP via mammalian target of rapamycin complex-1 (mTORC1) and results in accumulated cholesterol and FAs [68]. The knockdown and inhibition of SREBP1 and SREBP2 inhibits CRC cell proliferation and tumour growth in xenograft models [123].

Numerous studies have aimed to elucidate the cholesterol landscape in CRC. Increased levels of low-density lipoprotein (LDL) cholesterol have further been implicated in the risk of CRC development and progression [67]. Although one study showed no association of increased serum LDL-cholesterol in CRC recurrence after surgery [124], another group demonstrated higher LDL levels in CRC patients with distant hepatic metastasis and upregulated expression of LDL receptors (LDLR) in advanced CRC tumour tissues [67]. An accumulation of cholesterol was also observed in a study of 28 CRC tumour tissues when compared to normal adjacent tissues, and further showed increased LDLR expression by Western blot analysis [40]. A meta-analysis of 10 independent studies also showed an association of high total cholesterol with increased CRC risk and showed that high-density lipoprotein (HDL) cholesterol is associated with decreased CRC risk [125]. Intracellular cholesterol is esterified by O-acyltransferase 1/2 (SOAT1/2) and is commonly upregulated in CRC [69]. Xu et al. demonstrated that silencing SOAT1 in CRC cell lines promotes YAP expression via accumulation of cholesterol [69]. The role of cholesterol has recently been implicated in CRC liver metastasis [49]. SREBP2-dependent cholesterol biosynthesis was activated and required for CRC liver metastasis after orthotropic injection of HT29 CRC cells, such that SREBP2-dependent cholesterol biosynthesis was induced by the activation of the PI3K/mTOR/AKT pathway in the liver [49]. Additionally, the use of statins, which inhibits HMG-CoA reductase activity, has been associated with a reduced risk of developing CRC [126]. These studies may therefore provide therapeutic insights to the role of cholesterol in CRC tumorigenesis via the Wnt/YAP growth signalling pathway [65].

### 2.5. Glycerophospholipids

Glycerophospholipids (GP) comprise a family of amphiphilic lipids that contain various functional phosphate headgroups linked to a glycerol backbone containing FA moieties. The different functional headgroups include acid, glycerol, inositol, serine, ethanolamine, and choline moieties (Figure 1). Different headgroups drive distinct functions in a biological system (Table 1). For instance, phosphatidic acid (PA) species promote negative membrane curvature and membrane fusion, and also serve as important lipid precursor molecules [127]. Despite their naturally low abundance in animals, PA generated by the action of phospholipase D and diacylglycerol kinases have been implicated as second messenger molecules. Phosphatidylglycerols (PG) are typically present at 1–2% of animal tissue and serve as a precursor molecule to cardiolipins (CL), a GP comprising two PG molecules linked by glycerol. It has been shown that the acidic headgroups of PGs are important in anti-inflammatory processes [128]. Phosphatidylinositol (PI) lipids are typically low in abundance as biological membrane lipids; however, PI and PI-derived phosphoinositides have vital functions in protein interface binding, and as membrane trafficking and signalling molecules regulating signal transduction pathways [129,130]. Phosphatidylserine (PS) lipids account for up to 10% of total cellular phospholipid predominantly in the inner membrane leaflets [131]. PS have diverse roles in regulating apoptosis, blood clotting, and anti-inflammation processes [132]. Phosphatidylethanolamine (PE) and phosphatidylcholine (PC) lipids constitute the major GPs abundant in mammalian membranes. The cone-shaped PE lipids can modulate membrane fusion and stabilisation of membrane proteins [133]. PCs represent the major phospholipid in mammalian membranes, particularly in outer leaflets due to their cylindrical shape, and play roles in membrane integrity, rigidity, and mediating protein interactions [134]. In addition to the diverse functions driven by different phosphate headgroups, the fatty acyl and chain linkages that GPs bear results in increased structural diversity. GPs comprise diacyl, alkyl, alkenyl, and lyso acyl forms all with distinct biological functions (Figure 1).

Membrane GPs such as PCs and Pes are increased in CRC [55] to meet the demands of growing cells [2,55]. PC and PE lipids are also reported to be enriched with saturated FAs (SFA), resulting in more rigid membrane dynamics and correlating with poor prognosis in cancer patients [2]. Certain MUFA-containing PCs were also reported to be elevated in CRC tissue and five other cancerous tissue, including PC(32:1), and PC(34:1), while PUFAs and PUFA-containing polar lipids such as PC(38:4) and PE(38:4) were decreased, which increased the MUFA-to-PUFA ratio [46]. Another study demonstrated the potential of using a panel of lipids, including FAs and lysophosphatidylcholines (LPCs), to be used as CRC progression biomarkers [135]. Using a mass spectrometry-based imaging technique, elevated concentrations of PC(16:0/16:1) [136], LPC(16:0), LPC(18:1), and PC(16:0/18:1) [137] have been reported in CRC tissue compared to adjacent normal regions. Furthermore, other polar lipids have been shown to serve as potential biomarkers for CRC malignancy. For example, the level of PG(18:0/16:0) was elevated in the plasma of CRC patients, in contrast to a decreased level of LPC(18:3), LPC(18:2), PE(18:2/18:1), and PE(18:1/20:2) [138]. In patient-derived tissues, LPC(24:0), LPE(16:1), LPE(18:2), LPG(24:0), LPG(21:0), PS(16:0), PS(28:2), PG(37:6), PE(28:1), PE(31:4), PE(33:4), PC(26:0), PC(28:1), PC(30:0), PC(31:2), PC(31:4), PC(32:1), and PC(34:1) were all upregulated compared to tumour-adjacent tissues [39]. Other changes in GPs were also observed in exosomes derived from CRC patient plasma, namely, decreased PC(36:1) and increased PC(34:2), PC(36:2), PE(38:4), and PI(38:4) compared to healthy controls [47]. Furthermore, the sum ratio of 34:1-/38:4-containing GPs was lower in CRC exosomes compared to healthy controls, which demonstrates the propensity for PUFA-containing GPs in CRC exosomes as potential biomarkers [47]. Additionally among other cancers, an increase in lysophosphatidylcholine lipids has been reported in non-small cell lung cancer and proposed as early indicators of tumorigenesis [139].

In addition to changes in PC, a recent study of HT29 CRC cells showed an increase in PUFA-containing PA lipids such as PA(20:5) and PA(20:4) when treated with the approved CRC drug oxaliplatin [140]. A study of CRC tissues also revealed increased abundance of PA(36:2) and decreased levels of PA(38:3) and PA(40:5) [46]. Lysophosphatidic acid (LPA) interacts with receptors LPA1-3, and the expression of LPA1 and LPA2 have been reported to be dysregulated in CRC [141]. Transcriptomic analysis of CRC and normal mucosa showed decreased and increased mRNA expression levels of LPA1 and LPA2 in CRC, respectively [141]. These changes in phospholipids demonstrate the impact of membrane lipids in facilitating cancer cell metabolism.

Plasmalogen GPs are an important component of lipid raft microdomains involved in cellular signalling [142]. Plasmalogen lipids contain a *cis*-vinyl ether at the *sn*-1 and an ester bond at the *sn*-2 positions of the glycerol backbone, imparting molecule antioxidative properties that can protect lipids and lipoproteins from oxidative stress [143]. The structural and functional roles of ether lipids in cellular processes, which include membrane trafficking, cell differentiation, cell signalling, and antioxidant properties have been well described in the literature [142,144,145]. Indeed, the early study by Snyder and Wood in 1969, showing higher alkyl-ether neutral glyceride abundances in colonic tumour than normal tissue, was among the first reports of lipid alterations in cancer [146]. The plasmalogen subclass of glycerophospholipids regulate many aspects of cell membrane homeostasis, such as membrane fluidity, vesicle formation and ion transport and are also involved in signal transduction, cell proliferation, and reducing oxidative stress. Thus, ether lipids have gained interest as cancer therapeutic targets [142,145]. For instance, plasmalogen lipids were demonstrated as potential prognosis biomarkers for CRC metastases [142]. A recent study has further demonstrated deregulated levels of both alkyl-ether and alkenyl-ether plasmalogen in CRC. The alkenyl-ether PA(P-39:1) and alkyl-ether PA(O-38:1), PC(O-36:4), and PC(O-37:2) were all upregulated in CRC tissues relative to tumour-adjacent tissues [39]. Wang et al. further described increased abundance of PE(O-32:2), PE(O-32:1), PE(O-34:4), PC(O-32:0) in CRC tumours [45].

Although the role of ether lipids in CRC is relatively poorly understood, ether lipids, and the rate-limiting enzyme involved in ether lipid biosynthesis, alkylglycerone phosphate synthase (AGPS), have been reported to be elevated in breast tumour tissue compared to normal tissue [147]. More importantly, it has been demonstrated that AGPS downregulation in breast cancer cell lines lead to the attenuation of some tumorigenic properties such as cell survival, aggressiveness, and growth by altering not only ether lipid levels but other additional oncogenic signalling lipids, such as fatty acids, eicosanoids and lyso-glycerophospholipids [147]. The emerging evidence of dysregulated ether lipid metabolism in CRC indicates the necessity for further research in characterizing the role for plasmalogens and alkyl-ether lipids in CRC tumorigenesis.

The mitochondrial-specific CL lipids account for approximately ~15–20% of total phospholipids in the mitochondrial membrane [148]. CLs play a vital role in mitochondrial function and homeostasis. For example, respiratory chain super-complexes and OXPHOS enzymes associate with CL for optimal function. Respiratory Complex II links both the tricarboxylic acid (TCA) cycle and electron transport chains, and genetic aberrations in enzymes associated with Complex II have been linked to colon and other gastric cancers [148]. In HCT116 colon cancer cells, the depletion of oncogenic KRAS, or both hypoxia inducible factors-1α and -2α (HIF-1α, and HIF-2α, respectively) decreased the expression of mitochondrial phospholipid synthetic enzymes: ACSL5, PCK2, and AGPAT7 [48]. Furthermore, deregulation of these enzymes, particularly ACSL5 (acyl-CoA synthetic enzymes), have been shown to result in decreased CL content and impaired mitochondrial respiration.

## 3. Lipid-Associated Hallmarks of CRC

**Figure 2:**
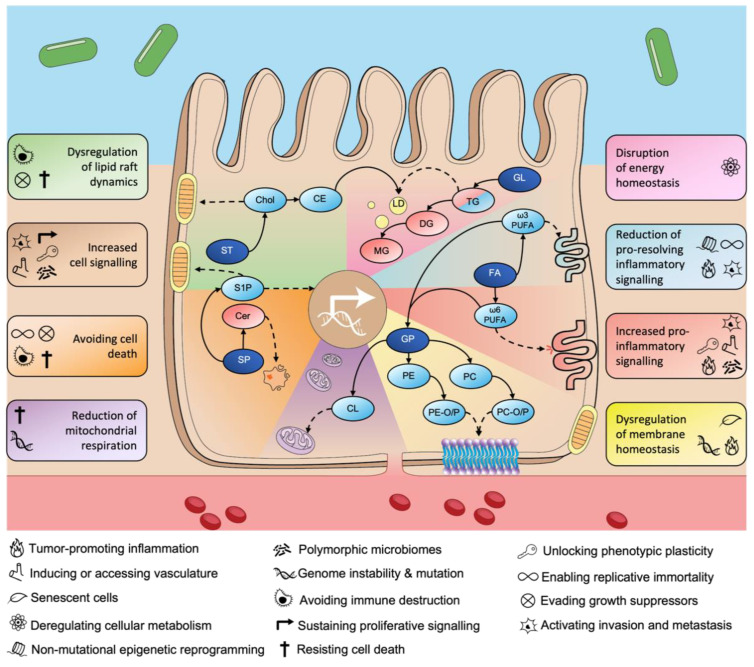
Reprogrammed lipid metabolism and the lipid-associated hallmarks of CRC. Reprogrammed lipid metabolism changes in CRC can be summarized into eight fundamental processes proposed here as the lipid-associated hallmarks of CRC (coloured boxes), each of which may be linked to one or more of the established cancer hallmarks (black icons) described by Hanahan and Weinberg [10,11,12]. Lipid categories are annotated as dark-blue ellipses, upregulated lipid classes are annotated in light-blue ellipses, downregulated lipid classes are annotated in red ellipses, solid black lines represent directly related lipid(s), and dotted black lines represent lipids involved with the lipid-associated hallmarks of CRC (icons). FA, fatty acid; GL, glycerolipid; GP, glycerophospholipid; ST, sterol; SP, sphingolipid; Chol, cholesterol; CE, cholesteryl-ester; Cer, ceramide; S1P, sphingosine-1-phosphate; PC, phosphatidylcholine; PC-O/P, ether-containing PC lipids; PE, phosphatidylethanolamine; PE-O/P, ether-containing PE lipids; CL, cardiolipin; TG, triacylglyceride; DG, diacylglyceride; MG, monoacylglyceride; LD, lipid droplet; PUFA, poly-unsaturated fatty acid.

Despite increasing reports of reprogrammed lipid metabolism in CRC, the direct associations between these changes and the established cancer hallmarks defined by Hanahan and Weinberg [11] have not yet been detailed. Here, we propose eight fundamental lipid metabolism-associated hallmarks of CRC, namely, (i) increased cell signalling, (ii) increased pro-inflammatory signalling, (iii) reduction in pro-resolving inflammatory signalling, (iv) disruption of energy homeostasis, (v) dysregulation of lipid raft dynamics, (vi) avoiding cell death, (vii) reduction in mitochondrial respiration, and (viii) dysregulation of membrane homeostasis (Figure 2). We also describe how each of these lipid-associated hallmarks may be mapped to the broader hallmarks of cancer defined by Hanahan and Weinberg.

### 3.1. Increased Cell Signalling

Increased cell signalling is a common cellular process occurring in CRC that encapsulates properties of multiple established cancer hallmarks including sustained proliferative signalling, inducing or accessing vasculature, activated invasion and metastasis, unlocking phenotypic plasticity, and polymorphic microbiomes (Figure 2). Changes in S1P, PA/LPA, and PGE2 metabolism allow CRC cells to gain increased cell signalling capabilities important for growth, invasion and metastasis, and angiogenesis). The accumulation of S1P can directly stimulate cell growth by activating PI3K, AKT, and MAPK signal transduction pathways [34], thereby sustaining proliferative signalling. S1PR1 has also been reported to be increased in CRC tissues and is highly associated with invasion, hepatic metastasis, and is positively correlated with poor patient survival [149]. Upregulated synthesis of PGE2 via S1P accumulation can activate invasion and metastasis by stimulating COX2-mediated EMT [79]. Lipid-associated changes can stimulate cell signalling crucial for the induction of angiogenesis; for example, LPAs induce the secretion of angiogenic factors in CRC cells [141], S1PR1 is essential for vascular barrier functions [7], and PGE2 has been shown to induce vascular endothelial growth factor (VEGF) [79,150]. Furthermore, down-regulation of the differentiation-promoting transcription factor SMAD family member 4 (SMAD4) in vivo resulted in WNT-mediated adenoma formation in differentiated mouse intestinal tissue [151]. SMAD4 is activated by bone morphogenetic protein (BMP) signalling [152], and although no associations have yet linked lipid metabolism and BMP signalling in CRC, BMP signalling was reported to regulate lipid metabolism and regulate mTORC1 signalling in hepatocytes [153]. ACSL4 was also reported to be a downstream target of BMP4 in a non-small-cell lung carcinoma model [154]. Complex GSLs interact with plasma membrane-bound receptors important in cell fate [155], and the propensity for differentiated colon-like cells to express unique GSLs and related glycosyltransferases potentiates a role for GSL glycans in cell differentiation [104]. Moreover, a recent study also showed Gb3Cer and Gb4Cer species were upregulated in dedifferentiated CRC tumour sites [156]. Polymorphic microbiomes are believed to modulate processes involved in growth, immune evasion, inflammatory response, and drug resistance [12]. As such, the imbalance of microbes, termed dysbiosis, in the gut has been linked to CRC risk and progression [157]. A high fat diet (HFD) and the gut microbiota showed vital significance as promoters of tumorigenesis In a murine model of azoxymethane (AOM)-induced CRC [158]. Faecal microbial transfer of HFD-AOM mice to germ-free mice resulted in dysbiosis, gut barrier dysfunction, and increased CRC development [158]. Metabolomic enrichment analysis also revealed that glycerophospholipid metabolism was significantly altered in HFD mice, and in particular, LPC and LPA lipids were upregulated in HFD mice [158], which suggests a role for dysbiosis in increased cell signalling in CRC.

### 3.2. Increased Pro-Inflammatory Signalling

Increased pro-inflammatory signalling is a characteristic process observed in CRC and can be associated with the cancer hallmarks tumour-promoting inflammation, inducing or accessing vasculature, activating invasion and metastasis, unlocking phenotypic plasticity, and polymorphic microbiomes (Figure 2). Stimulation of the pro-inflammatory response is common across various cancers, and changes to the metabolism of ω-6 PUFAs and their derivatives are observed in CRC, which leads to the stimulation of pro-inflammatory response [56,57] (Figure 2). The upregulation of ω-6 PUFAs and S1P in CRC leads to an accumulation of PGE2, a product vital for EMT transformation and VEGF function that stimulates the formation of new blood vessels [79]. PGE2 can also activate inflammatory immune cells required for progression and metastasis. For instance, PGE2 has been reported to modulate dendritic cell (DC) differentiation and function and promote chronic inflammation [159], upregulate interleukin-1 (IL-1) expression crucial for cell migration and VEGF function [150], and induce mast cell-derived secretion of proangiogenic factor MCP-1 [160]. Treatment of differentiating colon FHC cells with ω-6 AA and SCFA sodium butyrate stimulated an increase in differentiation activity, indicating a role for FAs in regulating cellular plasticity [161]. SCFAs have anti-inflammatory functions [162], and inhibit CRC cell growth [163], which suggests that SCFAs particularly derived from gut microbiota [164] may reduce CRC progression and drive colonic epithelia towards terminal differentiation. 

### 3.3. Reduction in Pro-Resolving Inflammatory Signalling

The inhibition of anti-inflammatory (pro-resolving) signalling is widely observed in CRC and is associated with multiple established hallmarks, namely, tumour-promoting inflammation, activating invasion and metastasis, enabling replicative immortality, and non-mutational epigenetic reprogramming (Figure 2). Dysregulated lipids that play crucial roles in reducing pro-resolving inflammatory signalling observed in CRC include ω-3 PUFAs and their derivative prostaglandins (Figure 2). Telomerase enzymes protect and maintain chromosomal telomere length, whereby its shortening causes senescence and cell growth arrest [165]. The ω-3 EPA and DHA lipids were upregulated in a study of CRC tissues [55]. EPA and DHA have shown efficacy in inhibiting telomerase activity in DLD1 CRC cells via human telomerase reverse transcriptase (*hTERT)* and *c-Myc* mechanisms, thus modulating the capacity for replicative immortality [166]. By reducing anti-inflammation, lipid-associated changes in CRC also induce invasion and metastasis. For instance, prostaglandin D2 and its receptor DP2 have been implicated with poor prognosis in CRC patients, where its upregulation in CRC is associated with VEGF expression [167]. Lipid-associated changes involved in non-mutational epigenetic reprogramming can be linked to changes in fatty acids that regulate anti-inflammatory response. For instance, tumour development was significantly reduced in rats fed with ω-3 PUFAs compared to controls, an effect attributed to a decrease in ω-6/ ω-3 PUFA ratio and increase in CpG epigenetic modification of cytosine to 5-methylcytosine (5mC) [168]. A recent study also showed that HCT116 and Caco2 CRC cells supplemented with ω-3 DHA resulted in the demethylation and expression of the miR-126 promoter, which correlated with decreased VEGF expression [169]. Another study showed that DHA increased 5mC levels in HCT116 compared to controls; however, DHA and EPA treatment resulted in a decrease in methylated genes. Lastly, EPA and DHA were shown to decrease expression levels of VEGF and COX2 and the abundance of PGE2 in HT29 CRC cells [170].

### 3.4. Disruption of Energy Homeostasis 

Reprogramming of lipids involved in energy metabolism is observed in CRC [99], and this disruption of energy homeostasis is comparable to the existing cancer hallmark of deregulated cellular metabolism [11]. Tumour cells accumulate TGs due to upregulated FASN and enhanced lipogenesis (Figure 2), and LD-resident TGs can be used for bioenergetic β-oxidation [83]. CRC stem cells contain accumulated LDs compared to normal epithelial colon cells [99]. As mentioned above, the dysregulation of TG and LD metabolism is omnipresent across various cancers and these alterations have been associated with the ability of cancer cells to utilize stored energy under conditions of nutrient-deprivation to promote cell survival [7]. The fatty acid translocase CD36, which has an integral role in the uptake of extracellular FAs and thus TG metabolism, is overexpressed in CRC tissue compared to normal colonic mucosa [84]. A recent study showed that FASN inhibition in CRC cells resulted in an upregulation of CD36 as a compensatory mechanism for FASN inhibition and promoted cell growth [84].

### 3.5. Dysregulation of Lipid Raft Dynamics

This CRC lipid hallmark can be associated with the established cancer hallmarks resisting cell death, evading growth suppressors, and avoiding immune destruction (Figure 2). Lipid rafts directly regulate signal transduction pathways by serving as platforms for signalling proteins, and changes to both membrane raft lipids cholesterol and sphingolipids have been implicated in cancer [121]. Alterations in cholesterol observed in CRC include the upregulation of ABCA1, which results in poor CRC prognosis via cholesterol transport mechanisms [66], and common *KRAS* and *PIK3CA* mutations that lead to accumulation of cholesterol to favour cell proliferation via SREBP activation [68]. Reprogramming of lipid metabolism that leads to the imbalance of ceramides and S1P also contribute to the dysregulation of lipid raft dynamics. A recent study demonstrated the efficacy of Cedrol in inhibiting HT29 CRC cell growth by destabilising lipid rafts through the redistribution of cholesterol from the plasma membrane, blocking Akt/mTOR signalling and inducing mitochondrial intrinsic apoptosis, thereby resisting cell death [171]. In a study of colonic cancer stem cell (CSC), impairment of cholesterol biosynthesis by deletion of key synthetic enzymes *HMGCR* or *FDPS* lead to reduced spheroid tumorigenic potential via activated TGF-β signalling [78], a key growth evasion pathway in cancer [7]. Lastly, disruption to membrane lipid raft dynamics via cholesterol may protect cancer cells from immune destruction. For instance, excess cholesterol levels characteristic of obesity impaired the development and function of natural killer T cells (NKT) and γδ T cells by hematopoietic stem cells, which resulted in increased CRC risk in mice [172].

### 3.6. Avoiding Cell Death

Avoiding cell death is a key hallmark of cancer [7]; however, lipid changes in CRC leading to this process can also be associated with other hallmarks, including enabling replicative immortality, evading growth suppressors, resisting cell death, and avoiding immune destruction (Figure 2). Alterations to ceramide metabolism in CRC leads to processes that favour cancer cell survival [42,173]. Reports of differential expression of ceramides between CRC and non-cancerous tissues indicate a reprogramming of ceramide metabolism in CRC [42,108]. Ceramides are considered anti-apoptotic molecules by causing FAS receptor aggregation within lipid rafts, thereby inducing apoptosis [173]. C16 ceramide is increased in CRC [108], which binds the tumour suppressor p53 and inhibits E3 ligase-mediated ubiquitination of p53 [174]. This in turn prevents canonical death signalling pathways downstream of p53 [174]. Although no link has been implicated for ceramide changes in CRC and enabling replicative immortality, C6 ceramide was shown to inhibit telomerase activity in A549 lung cancer cells by inducing G0/G1 cell cycle arrest [175]. Additionally, accumulation of C18 ceramide was also shown to promote replicative immortality in A549 cells by inducing the deacetylation of transcription factor Sp3, which inhibited the *hTERT* promoter [176].

### 3.7. Reduced Mitochondrial Respiration 

This lipid-associated CRC hallmark can be linked to the cancer hallmarks genome instability and mutation, and resisting cell death [11] (Figure 2). CLs are central to this CRC hallmark as they are vital regulators of mitochondrial function and apoptosis [177]. PUFA-containing CLs are highly prone to lipid peroxidation, which results in the formation of superoxide products such as 4-HNE that facilitate the activation of apoptotic machinery [178]. Furthermore, CLs aid in the stabilisation of OXPHOS complexes within the inner mitochondrial membrane and promote optimal cellular respiratory function [148]. In CRC, loss of KRAS in HCT116 cells downregulated the expression of mitochondrial synthetic enzymes and caused a reduction in CL content leading to mitochondrial respiration dysfunction [48]. Lipid peroxidation of CL within the mitochondria leads to the formation of 4-HNE, which can cause DNA damage, prevent electron transport chain activity, and decrease TCA cycle activity [179]. As CL metabolism is poorly characterized in CRC, additional research efforts in this area may provide a better understanding of mitochondrial-associated changes in CRC and provide beneficial therapeutic strategies.

### 3.8. Dysregulation of Membrane Homeostasis

Lipid changes in CRC that lead to dysregulated membrane homeostasis are associated with multiple cancer hallmarks, namely, promoting inflammation, genome instability and mutation, and cell senescence (Figure 2). The interaction between ROS and unsaturated lipids give rise to lipid peroxidation and products, such as 4-hydroxy-nonenal (4-HNE), that can modulate protein and lipid function, and induce DNA damage [179]. Membranous plasmalogens are highly prone to oxidation at the *sn-1* position, thereby preventing the oxidation of adjacent PUFA-containing lipids [142]. Investigations of the plasmalogen content of CRC tissues and plasma has implicated the dysregulation of plasmalogen lipid metabolism in CRC [45,57,144]. CRC cases often involve mutations in the genes such as *APC*, *TP53*, *KRAS*, and *PI3KCA* [18]; however *TP53* has previously been associated with the hallmark genome instability and mutation [7]. The dysregulation of plasmalogen lipids in CRC may be implicated in genome instability and mutation as lipid peroxidation products such as 4-HNE have been shown to form adducts with the gene *TP53*, causing impaired biological function [179]. Plasmalogen PEs have been shown to suppress apoptosis in CACO-2 CRC cells [180]. This anti-apoptotic activity was associated with inhibition of downstream inflammatory responses that promote cell death [180]. Senescent cells proceed into a senescence-associated secretory phenotype (SASP) and can provide the TME with pro-tumorigenic factors beneficial for cancer cell growth and proliferation [12]. Phospholipase D (PLD) functions to produce PA and choline from PC and to regulate membrane homeostasis and protein transport [63]. PLD2 mRNA was upregulated in CRC tumours, and primary fibroblasts cultured in media used to grow PLD2-overexpressing CRC cells showed increased senescence [63]. This study demonstrated that PLD2 and its product PA secreted by CRC cells can induce senescence of neighbouring fibroblasts and accumulate SASP factors to maintain cancer cell stemness. The secretion of growth differentiation factor 15 (GDF15), a pro-tumorigenic SASP factor, was induced in senescent colon fibroblasts, which activated MAPK and PI3K signalling [77]. In a study of fibrosarcoma-bearing mice, inhibition of GDF15 function was reported to reverse lipid oxidation and prevent cachexia [181], posing a currently unexplored association of SASPs such as GDF15 in mediating membrane lipid oxidation in CRC.

## 4. Conclusions

The diverse structures of lipids provide these molecules with multifarious functions vital for cellular homeostasis. An increasing body of evidence supports the notion that reprogrammed lipid metabolism involving remodelling of cellular membrane structure, and changes in energy homeostasis and signalling of cancer-associated pathways, plays a pivotal role in the onset, progression and maintenance of CRC and their metabolic tumour properties. Despite progress in our understanding of cancer cell metabolism and lipidomics in the last ten years, the hallmarks of cancer, which describe the concomitant aberrant cellular processes believed to unlock the uncontrollable proliferative nature of malignant cells, have yet not been explicitly associated with lipid-associated changes in CRC. Here, by linking the established hallmarks of cancer to recent reports of reprogrammed lipid metabolism in CRC, we propose eight fundamental processes as lipid-associated hallmarks of CRC.

## Figures and Tables

**Table 1:** Functional characteristics of lipid molecules and associated enzymes implicated in CRC. The diverse functions of lipids and associated enzymes implicated in CRC can be broadly categorized into three key processes: membrane structure, cell signalling, and energy homeostasis.

Function	Membrane Structure	Cell Signalling	Energy Homeostasis
Lipids	PC, PE, PS [39] Chol [40] S1P [41], Cer [42], SM [43] SFA [44]	PC, PE, PS, PG, LPC, LPE, PA-O, PC-O [39], PE-O [45], PA [46], PI [47], CL [48] Chol [49] S1P [50,51], Cer [42], SM [43] MUFAs [52,53,54], PUFAs [55,56,57]	TG [58], DG, MG [39] CL [48] CE [59] Acyl-CoA [60], VLCFA [61,62]
Enzymes	Phospholipase-D 2 (PLD2) [63] Stearoyl-CoA desaturase 1 (SCD1) [50,51,52] Serine/threonine-protein kinase PIM-3 (PIM3) [64] HMG-CoA reductase (HMGCR) [65] ATP-binding cassette transporter 1 (ABCA1) [66] LDL-receptor (LDLR) [40,67] Sterol regulatory element binding protein (SREBP) 1-2 [68] Sterol O-acyltransferase (SOAT) 1-2 [69] Ceramide synthase (CERS) [70,71] Ceramidase (CDase) [72] Alkaline-sphingomyelinase (Alk-SMase) [73,74] S1P phosphatase (SPP) 1-2 [75] Sphingosine kinase (SK) 1-2 [75] S1P lyase (SPL) [75] Fatty acid synthase (FASN) [24,76] FA-elongase (ELOVLs) [61] FA-desaturase (FADS) 1-2 [58]	Lysophosphatidylcholine acyltransferase-2 (LPCAT2) [59] Phospholipase-D 2 (PLD2) [63] Phosphoinositide 3-kinase (PI3K) [77] Stearoyl-CoA desaturase 1 (SCD1) [50,51,52] Serine/threonine-protein kinase PIM-3 (PIM3) [64] HMG-CoA reductase (HMGCR) [78] ATP-binding cassette transporter 1 (ABCA1) [66] LDL-receptor (LDLR) [40,67] Sterol regulatory element binding protein (SREBP) 1-2 [49,68] Ceramide synthase (CERS) [70,71] Ceramidase (CDase) [72] Alkaline-sphingomyelinase (Alk-SMase) [73,74] S1P phosphatase (SPP) 1-2 [75] Sphingosine kinase (SK) 1-2 [75] S1P lyase (SPL) [75] Fatty acid synthase (FASN) [24,76] FA-elongase (ELOVLs) [52,54] FA-desaturase (FADS) 1-2 [58] Acyl-CoA synthetase (ACSL) 4-5 [48,60] Cyclo-oxygenase 2 (COX2) [79]	Adipose triglyceride lipase (ATGL) [80] Monoacylglycerol lipase (MGL) [81] Hypoxia inducible factor (HIF) 1-2α [48] Sterol O-acyltransferase (SOAT) 1-2 [69] Stearoyl-CoA desaturase 1 (SCD1) [50,51] Acyl-CoA synthetase (ACSL) 1,4,5 [48,60,82] FA-elongase (ELOVLs) [62] Fatty acid synthase (FASN) [83] Cyclo-oxygenase 2 (COX2) [79] FA-translocase CD36 (CD36) [84] FA-desaturase (FADS) 1-2 [58]

Lipids and enzymes are denoted in colour as a function of lipid category. FA (orange), fatty acyl; GL (green), glycerolipid; GP (blue), glycerophospholipid; ST (red), sterol; SP (purple), sphingolipid; Chol, cholesterol; CE, cholesteryl-ester; Cer, ceramide; S1P, sphingosine-1-phosphate; SM, sphingomyelin; PC, phosphatidylcholine; PC-O, alkyl-ether PC lipid; LPC, lysophosphatidylcholine; LPE, lysophosphatidylethanolamine; PE, phosphatidylethanolamine; PE-O, alkyl-ether PE lipid; PA, phosphatidic acid; PA-O, alkyl-ether PA lipid; PS, phosphatidylserine; PI, phosphatidylinositol; PG, phosphatidylglycerol; CL, cardiolipin; TG, triacylglyceride; DG, diacylglyceride; MG, monoacylglyceride; PUFA, poly-unsaturated fatty acid; MUFA, mono-unsaturated fatty acid; SFA, saturated fatty acid; VLCFA, very long-chain fatty acid.
